# Zinc and Depression: From Neurobiological Functions to Gut–Brain Interactions

**DOI:** 10.3390/nu18162584

**Published:** 2026-08-07

**Authors:** Haichuan Li, Jiazhang Huang, Tian Zhang, Zhenchuang Tang, Qirui Xu, Di Gong, Liang Wang, Ying Zhang

**Affiliations:** 1 School of Public Health, Lanzhou University, Lanzhou 730030, China; 2 Institute of Food and Nutrition Development, Ministry of Agriculture and Rural Affairs, Beijing 100081, China; 3 Department of Public Health, Global Health Institute, Marshall University, Huntington, WV 25703, USA

**Keywords:** zinc, depression, gut microbiota, probiotics, zinc supplementation

## Abstract

The global prevalence of depression and its profound burden on public health create an urgent need to explore novel interventions beyond conventional therapeutic strategies. Zinc, a key regulator in the central nervous system, exerts multiple biological functions beyond maintaining neurotransmitter homeostasis, including inhibiting neuroinflammation, restoring neuroplasticity, and modulating gut microenvironmental homeostasis. This review summarizes the evidence linking zinc deficiency to the onset and development of depression, with a specific focus on the role of gut microbiota. It also discusses different forms of zinc supplements, highlighting synergistic interventions that combine novel zinc complexes with probiotics. Together, these lines of evidence provide a theoretical basis for innovative adjunctive strategies for depression.

## 1. Introduction

Depression is a highly prevalent mental disorder that severely impairs patients’ quality of life and social functioning, making it a major public health concern. According to the World Health Organization (WHO), depression affects approximately 8.4% of the global adult population and is projected to become the second leading cause of disease burden by 2030 [1]. Clinically, individuals with depression commonly present with persistently low mood, loss of interest in activities, and cognitive impairment. These symptoms impose a heavy burden on patients, their families, and society at large [2].

In recent years, the multifaceted physiological roles of micronutrients—particularly zinc—within the nervous system have become increasingly clear. As a key modulator of neurotransmitters, zinc contributes to synaptic transmission, neuroprotection, and neuroplasticity [3]. Several studies have revealed a strong association between zinc deficiency and the onset of depression [4]. Depressed patients often exhibit lower zinc levels, and zinc supplementation has been shown to alleviate depressive symptoms [4], suggesting that zinc may serve as both a potential biomarker and an adjunctive treatment for depression. Furthermore, zinc influences multiple brain signaling pathways, including those involving brain-derived neurotrophic factor (BDNF) and inflammatory responses, thereby mediating neurobiological alterations associated with depression [3].

The gut–brain axis, a critical bidirectional pathway linking the central nervous system to the gut microbiota, offers new insights into the relationship between zinc deficiency and depression. The gut microbiota influences mood and behavior by regulating neurotransmitter synthesis, immune-inflammatory responses, and neuroendocrine functions [5]. A growing body of evidence indicates that individuals with depression exhibit altered gut microbiota composition, and that interventions targeting the gut microbiota-including probiotics, prebiotics, and dietary modifications-can ameliorate depressive-like behaviors [6]. As a trace element, zinc participates in neurotransmitter metabolism and influences intestinal barrier function and microecological balance. Zinc deficiency may therefore promote the onset and progression of depression, at least in part, by disrupting the gut–brain axis [7]. Consequently, a critical narrative review examining the role of zinc in the pathophysiology of depression, with a particular focus on the microbiota–gut–brain axis, may yield valuable mechanistic insights.

## 2. Basic Properties of Zinc and Its Role in the Host Brain

### 2.1. Basic Properties of Zinc

Zinc has a common oxidation state of +2 and an electron configuration of [Ar] 3d^10^4s^2^, endowing it with strong coordination ability. It readily forms structural zinc ions through coordination with histidine and cysteine residues. These residues provide hydrogen-bond donor and acceptor groups that stabilize zinc-binding sites and promote protein folding and stability [8]. They also mediate interactions with other macromolecules.

The status of zinc in the human body is difficult to characterize; a property closely tied to its classification as a type 2 nutrient. Type 2 nutrients, such as zinc, nitrogen, and magnesium, are essential for multiple metabolic functions [9]. Unlike type 1 nutrients such as iron and thiamine, which serve specific roles, deficiencies in type 2 nutrients trigger more complex compensatory mechanisms. When zinc intake is insufficient, the body first substantially reduces endogenous losses [10]. Within days, endogenous fecal losses drop sharply, and during severe depletion, urinary excretion also declines [11]. If equilibrium cannot be restored, the body draws upon small, vulnerable zinc pools and may initiate tissue catabolism to release zinc [9]. For example, experimental animals fed low-zinc diets exhibit periodic feeding patterns, with muscle catabolism releasing zinc during starvation phases to prolong survival [12]. This same diagnostic challenge, the lack of a reliable tissue-level signal, also complicates clinical assessment of zinc deficiency. Because the body maintains only extremely limited zinc reserves, tissue zinc concentrations do not decrease substantially even during depletion. The absence of specific clinical or biochemical markers makes diagnosis difficult. Yet the response to zinc supplementation is remarkably rapid: enzyme resynthesis and reversal of skin lesions can occur within days [9,13].

Zinc is an essential trace element that performs a broad range of key functions in living organisms. As a cofactor for numerous enzymes, it participates in catalytic reactions, regulates enzyme activity, and stabilizes protein spatial conformation. It also plays a vital role in gene expression regulation: zinc ions bind to zinc finger motifs within proteins, contributing to transcription factor assembly and regulating gene transcription [14]. In addition, zinc acts as a signaling molecule in cellular pathways, influencing biological processes such as cell proliferation, differentiation, and apoptosis [15].

**Figure 1:**
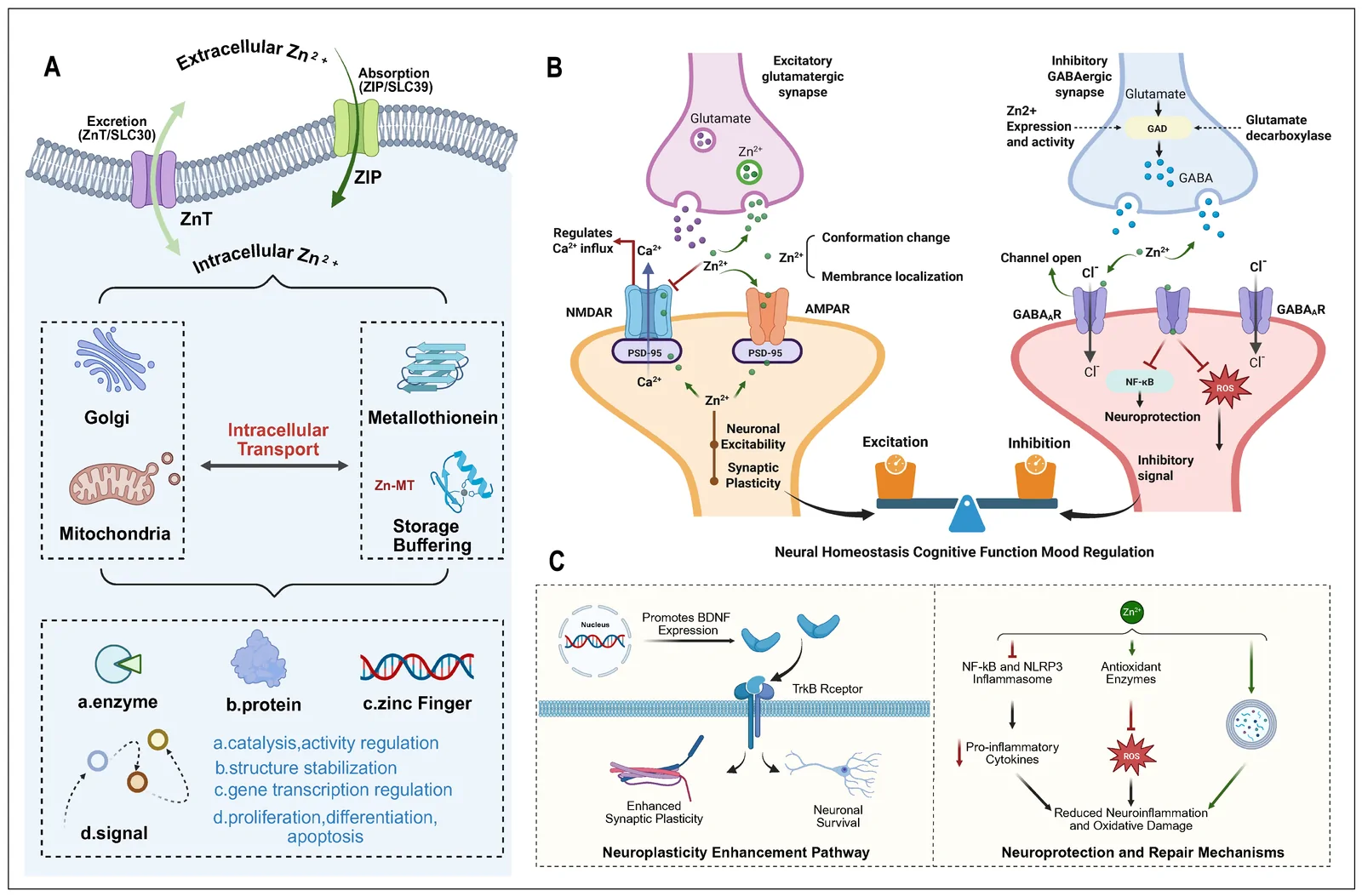
The fundamental physiological roles of zinc and cellular homeostasis mechanisms (**A**). The schematic diagram illustrates zinc’s dual regulatory role in excitatory and inhibitory neurotransmission (**B**). Zinc exerts multifaceted effects upon neural plasticity and neuroprotection, primarily by enhancing neuronal survival and synaptic plasticity through the promotion of BDNF expression, while concurrently mitigating neuroinflammatory damage via antioxidant and anti-inflammatory actions (**C**). Green arrows are used to denote promotion or activation, while red flat-headed arrows and red arrows are used to indicate inhibition or blockade.

As shown in Figure 1, zinc homeostasis in the body relies on a complex regulatory system governing absorption, storage, and excretion. Gastrointestinal zinc absorption is mediated primarily by two families of zinc transporters: the ZIP family (SLC39) for zinc uptake and the ZnT family (SLC30) for zinc efflux [16,17]. Through coordinated expression, these transporters maintain the equilibrium between intracellular and extracellular zinc concentrations [16,17]. Metallothioneins (MTs) function as zinc storage and buffering proteins, regulating the concentration of free zinc ions to prevent toxicity while supplying available zinc to cells [14]. Zinc metabolism also involves intracellular trafficking between organelles, including the Golgi apparatus and mitochondria, processes that are finely regulated by specific zinc transporters and regulatory factors [18,19].

### 2.2. The Role of Zinc in the Host Brain and Neurotransmitter Regulation

#### 2.2.1. The Role of Zinc in the Brain

As an essential trace element, zinc performs multiple functions within the nervous system, with a particularly crucial role in neurotransmitter regulation. It serves not only as an enzyme cofactor in diverse biochemical reactions but also as a regulator of neural signaling, contributing to the delicate balance between neural excitation and inhibition [20].

In excitatory neurotransmission, zinc exerts its effects primarily by modulating glutamate receptors, particularly *N*-methyl-D-aspartic acid (NMDA) and α-amino-3-hydroxy-5-methyl-4-isoxazolepropionic acid (AMPA) receptors [21]. NMDA receptors are pivotal in excitatory transmission, and their excessive activation is closely associated with various neuropathological states [22]. Zinc regulates NMDA receptor function in a concentration- and subtype-dependent manner, producing both inhibitory and modulatory effects [23]. Zinc also binds directly to NMDA receptor subunits, modulating channel gating [24]. Zinc likewise regulates AMPA receptor function and thereby rapid excitatory synaptic transmission. It does so by altering receptor conformation and membrane localization, which in turn modulates receptor-mediated currents, synaptic strength, and neural network activity [25]. Research shows that zinc released from zinc-enriched neurons in the hippocampus following synaptic activity modulates AMPA receptor-mediated signaling and contributes to the regulation of higher-order brain functions, including learning and memory [26].

Adequate zinc levels are essential for maintaining synaptic function and neuroplasticity. Zinc modulates neuronal excitability in a concentration-dependent manner: nanomolar concentrations suppress NMDA receptor-mediated excitatory transmission, reducing the risk of neurotoxicity [27]. Zinc also influences glutamate receptor localization and function by binding to membrane-associated proteins such as PSD-95, which provides an additional layer of regulation over excitatory synaptic transmission [28]. Collectively, zinc exerts multi-target regulation of glutamate receptors to finely tune excitatory neurotransmission and maintain neural homeostasis.

In addition to its role in excitatory neurotransmission, zinc also participates in regulating inhibitory neurotransmitter systems, particularly the gamma-aminobutyric acid (GABA) system [29]. GABA is the primary inhibitory neurotransmitter in the central nervous system, and tight regulation of its receptor function is essential for maintaining the balance between neural excitation and inhibition. Research indicates that zinc modulates neuronal inhibitory currents by binding to GABA_A receptor subunits, regulating receptor channel opening probability and sensitivity to GABA [30]. This regulatory role is important for modulating neuronal excitability thresholds and synaptic plasticity [20]. One study suggests that zinc influences glutamate decarboxylase activity in the hippocampus [31]. The impact of zinc on these enzymes may be shaped by intergenerational genetic regulation, although research in this area remains limited. For example, in a rat model of schizophrenia involving prenatal lipopolysaccharide exposure in the parental generation, zinc supplementation restored working memory deficits and GAD67 expression in male offspring [32]. Zinc also attenuates neuroinflammation and oxidative stress. In experimental systems, zinc can attenuate NF-κB signaling and reactive oxygen species production, which may protect GABAergic neuronal function. Whether these effects translate into clinically meaningful improvements in depression or anxiety remains uncertain [33].

#### 2.2.2. Neuroplasticity and Neuroprotection: The Role of Zinc

Experimental studies indicate that zinc can modulate BDNF/TrkB signaling, neuronal survival, and synaptic plasticity [34,35]. These pathways are mechanistically relevant to depression, but evidence that zinc-induced changes in BDNF mediate antidepressant effects in humans remains limited. Cellular and animal studies also suggest that zinc can influence inflammatory signaling, antioxidant enzyme activity, autophagy, and NLRP3 inflammasome activation [36,37,38,39]. These findings support potential neuroprotective actions of zinc in preclinical settings. In preclinical models of brain injury and neurotoxicity, zinc-containing interventions have promoted neuronal survival and functional recovery [36,40,41]. However, these models do not directly establish antidepressant efficacy, and both zinc deficiency and zinc excess can impair neural function [42].

## 3. Zinc and Depression: Evidence from Animal Models

The neurobiological actions described in Section 2 indicate that zinc contributes to the balance of excitatory and inhibitory neurotransmission, synaptic plasticity, neurotrophic support, and redox and inflammatory homeostasis. However, can altered zinc status lead to emotional and behavioral abnormalities? Animal studies have addressed this question by restricting dietary zinc intake, restoring zinc availability, combining zinc deficiency with depression-related models, and manipulating zinc-sensing pathways. Collectively, these approaches have provided insight into how disrupted zinc homeostasis translates molecular alterations into depression-related behaviors. The following sections discuss this evidence from several perspectives.

### 3.1. Dietary Zinc Deficiency and Depression-Related Behavioral Phenotypes

Dietary zinc restriction is one of the principal experimental approaches used to investigate the relationship between zinc homeostasis and depression-related behaviors. Tassabehji et al. fed adult male rats zinc-adequate, zinc-deficient, or zinc-supplemented diets for three weeks [43]. Compared with animals receiving the zinc-adequate diet, zinc-deficient rats exhibited reduced food intake, decreased preference for a saccharin solution, and increased anxiety-like behavior in the light–dark box test, suggesting that sustained zinc insufficiency may simultaneously affect feeding motivation, reward responsiveness, and stress-related behavior. In addition, fluoxetine reduced behavioral passivity in the forced swim test in zinc-adequate and zinc-supplemented animals but did not produce a comparable effect in zinc-deficient animals, indicating that baseline zinc status may also influence responsiveness to antidepressant treatment [43]. Short-term zinc deprivation can likewise induce behavioral and neurochemical alterations. In young rats, two weeks of exposure to a low-zinc diet increased immobility in the forced swim test, accompanied by changes in stress responses and hippocampal neurochemical markers [44]. However, the behavioral effects of zinc deficiency do not appear to increase in a simple linear manner with the duration of the intervention. A time-course study showed that mice maintained on a zinc-deficient diet for 2, 4, or 10 weeks displayed distinct behavioral responses in the forced swim test, with longer-term zinc restriction being more consistently associated with increased behavioral passivity and dysregulated stress responses [45]. Similar findings have been reported in other animal studies: zinc-deficient mice exhibited more pronounced depression-related behaviors and altered neural activity within limbic regions, whereas antidepressant treatment reversed some of the behavioral abnormalities and aberrant patterns of regional brain activation [46]. Collectively, these studies suggest that sustained zinc insufficiency may induce adaptive changes in the neural networks that regulate reward processing, stress responses, and emotional behavior. At the whole-animal level, these changes manifest as anhedonia, increased behavioral passivity, and heightened stress susceptibility.

### 3.2. Disrupted Zinc Homeostasis Induces Neurobiological Remodeling and Modifies Antidepressant Responses

In zinc-deficient rats, hippocampal expression of the NMDA receptor subunits GluN1, GluN2A, and GluN2B is increased, accompanied by reduced phosphorylation of the AMPA receptor subunit GluA1, decreased CREB activation, and lower BDNF expression. Chronic fluoxetine treatment improves behavior in the forced swim test and partially ameliorates the abnormalities in NMDA receptor and CREB signaling [47]. The effects of different antidepressant classes also diverge under zinc-deficient conditions. Zinc deficiency modifies the behavioral effects of escitalopram, imipramine, reboxetine, and bupropion in the forced swim test, with acute and chronic administration producing outcomes that are not always consistent [48]. In the olfactory bulbectomy model, zinc deficiency further exacerbates certain depression-related behaviors and is accompanied by alterations in zinc concentrations and zinc-homeostatic proteins in the serum, prefrontal cortex, and hippocampus. Individual antidepressants also vary in the extent to which they restore behavioral outcomes and tissue zinc homeostasis [49]. These findings suggest that baseline zinc status influences the neurotransmitter, receptor, and plasticity-related networks on which antidepressant drugs depend, thereby modifying the effects of specific antidepressants.

Studies of the zinc-sensing receptor GPR39 further indicate that zinc-related behavioral effects are mediated through specific signaling mechanisms. GPR39-knockout mice exhibit depression-related behaviors in the forced swim and tail suspension tests, together with reduced hippocampal expression of CREB and BDNF [50]. Consistent with these findings, the GPR39 agonist TC-G 1008 produces antidepressant-like effects in mice. Under the conditions tested, the effects of a single administration persist for up to 24 h, longer than those observed with zinc chloride, imipramine, or MK-801. However, the pharmacokinetic properties, receptor selectivity, and long-term safety of this compound require further investigation [51]. The antidepressant-like effects of TC-G 1008 are attenuated by NMDA receptor activation or GABA_A receptor blockade. A low-zinc diet is also associated with abnormal expression of GluN1, PSD95, and KCC2, and it diminishes the behavioral effects of TC-G 1008 under certain experimental conditions [52]. Beyond receptor-mediated and synaptic signaling, omics studies indicate that zinc deficiency may affect broader networks underlying brain function. Proteomic analyses of the prefrontal cortex and hippocampus of zinc-deficient rats identified alterations in several zinc transporters and mitochondrial proteins, together with reduced mitochondrial complex I activity in the prefrontal cortex [53]. These findings suggest that depression-related phenotypes arising from disrupted zinc homeostasis involve neurotransmission, synaptic plasticity, and cerebral energy metabolism simultaneously, rather than being driven by a deficit in any single neurotransmitter system.

### 3.3. Extending the Effects of Zinc from the Brain to Intestinal and Systemic Homeostasis

Although early studies focused primarily on neurotransmission and neural plasticity within the brain, dietary zinc deficiency originates in the gastrointestinal system and subsequently affects zinc absorption, tissue distribution, immune function, and redox status. The brain and behavioral abnormalities observed in animals may therefore reflect a broader disruption of systemic zinc homeostasis rather than an isolated reduction in central zinc availability. In rats maintained on zinc-adequate or zinc-deficient diets for 4–6 weeks, zinc restriction not only reduced zinc concentrations in the serum and selected brain regions but also induced redistribution of other bioelements, including iron. Zinc-deficient animals exhibited oxidative damage and increased levels of inflammatory markers, including IL-1α and IL-1β, in the prefrontal cortex and hippocampus, together with elevated extracellular glutamate concentrations in the prefrontal cortex [54]. These findings demonstrate that zinc deficiency simultaneously disrupts trace-element homeostasis, redox balance, inflammatory responses, and neurotransmitter release, linking peripheral nutritional status to alterations in central nervous system function. Studies of the host gastrointestinal system have further broadened this systemic perspective. In pregnant mice, zinc deficiency or reduced dietary zinc bioavailability altered the composition of the gastrointestinal microbiota, accompanied by abnormalities in intestinal barrier markers, increased hepatic lipopolysaccharide levels, and elevated cerebral expression of GFAP and IL-6. Supplementation with a zinc–amino acid complex partially reversed these changes in the microbiota, intestinal tissues, and inflammatory markers [55]. Accordingly, the relationship between zinc and depression-related phenotypes cannot be understood solely in terms of the direct regulation of brain receptors and neurotransmitters by zinc. Zinc is ingested and absorbed through the gastrointestinal tract and maintains intestinal epithelial integrity, microbial ecology, and immune homeostasis. When dietary zinc supply or bioavailability is reduced, abnormalities in central neurotransmission and neural plasticity may arise in parallel with intestinal barrier impairment, altered microbial composition, and peripheral inflammation. It is therefore necessary to consider whether the intestine and its microbial communities serve as a critical intermediary linking dietary zinc status to impaired central regulation of mood.

## 4. Zinc and Depression: A Potential Role of the Microbiota–Gut–Brain Axis

As discussed in Section 2 and Section 3, an imbalance in zinc homeostasis may be associated with changes in neurotransmitters, neuroplasticity, inflammatory states, and depression-related behaviors. Animal studies further indicate that two weeks of dietary zinc deprivation can increase stress susceptibility and depression-related behavior, accompanied by altered corticosterone responses and enhanced stimulus-evoked hippocampal glutamate release [56]. In rats, four to six weeks of zinc restriction was associated with behavioral despair, anhedonia, reduced social behavior, increased hippocampal GluN2A and GluN2B expression, and reduced PSD-95, *p*-CREB, and BDNF levels [57]. It is important to note that several studies have explicitly indicated that the gut microbiota is important in depression [58]. Based on this result, this chapter will discuss, in turn, the relationship between the microbiota-gut–brain axis and depression, and the effects of zinc status on the gut microbiota.

### 4.1. The Microbiota–Gut–Brain Axis in Depression

**Figure 2:**
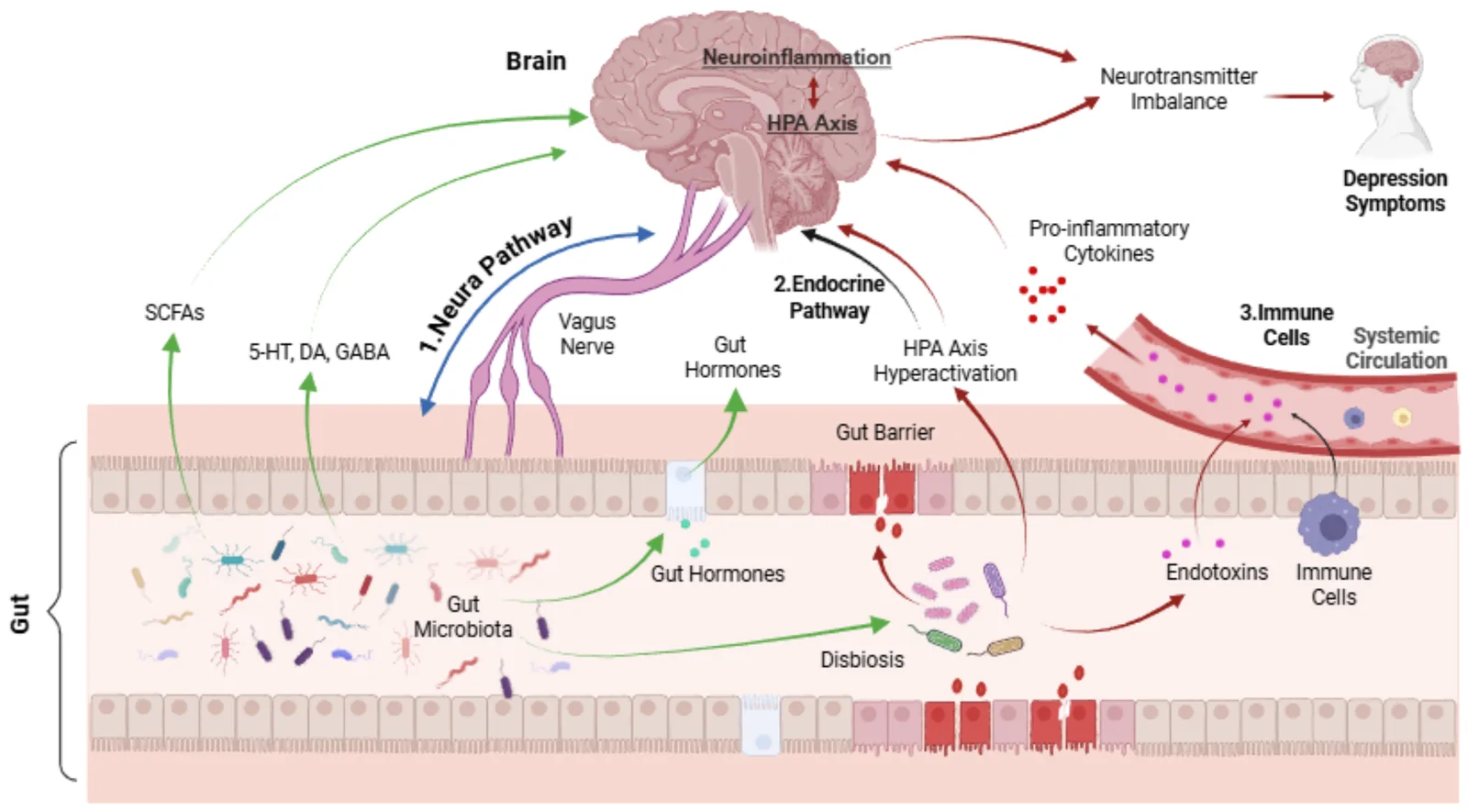
The following schematic diagram illustrates the intricate network that exists between the gastrointestinal tract and the brain, and its role in the development of depression.

The gut–brain axis represents a sophisticated network of bidirectional communication between the gastrointestinal system and the central nervous system through multiple physiological pathways. The nervous system is not the only system involved; the immune and endocrine systems are also implicated, and together they form a regulatory system that is multi-level and multi-pathway [58]. Communication along this axis occurs through neural, immune, and endocrine pathways [59]. Neural routes include the vagus nerve and the enteric nervous system, whereas immune and endocrine routes involve mucosal and systemic immune responses, gut hormones, and the hypothalamic–pituitary–adrenal axis (Figure 2).

The gut microbiota is a key component of the gut–brain axis and profoundly influences the onset and progression of depression. It does so by regulating neurotransmitter synthesis, modulating inflammatory responses, and shaping stress reactivity. Gut microbial metabolites, including short-chain fatty acids (SCFAs), neurotransmitter precursors, and related compounds, influence central nervous system function and emotional regulation [59]. Gut dysbiosis compromises intestinal barrier function, allowing endotoxins to enter the bloodstream and trigger systemic inflammatory responses [60]. The resulting inflammatory state activates neuroimmune pathways, disrupting neurotransmitter balance and promoting neuroinflammation, which in turn exacerbates depressive symptoms [59].

Gut microbiota synthesize several neurotransmitters, including serotonin (5-HT), dopamine (DA), and GABA [61]. Beyond this direct synthesis, the microbiota also influences central neurotransmission and emotional states indirectly by regulating the release of gut hormones from enteroendocrine cells [62]. For example, the 5-HT system is implicated in the comorbidity of depression and gastrointestinal disorders, and 5-HT4 receptor agonists show potential for alleviating depressive symptoms and improving associated intestinal dysfunction [3]. Gut microbiota also influences stress adaptation by modulating hypothalamic–pituitary–adrenal (HPA) axis activity. Excessive HPA axis activation is a key biological mechanism underlying the onset of depression [4,5]. In recent years, researchers have focused increasingly on the interaction between gut microbiota and gut hormones [60]. Gut hormones not only regulate gastrointestinal function but also serve as signaling molecules that convey information along the gut–brain axis. Their release is modulated by gut microbiota, which in turn shape neuroendocrine function and behavior, offering new targets for depression treatment [60].

Population-based microbiome studies provide evidence linking the gut microbiota to depression. Valles-Colomer et al. analyzed the gut microbiome of 1054 participants and found that butyrate-producing bacteria were associated with higher quality of life, while *Coprococcus* and *Dialister* were relatively depleted in individuals with depression [63]. Radjabzadeh et al. examined the association between gut microbiota and depressive symptoms in 1133 participants from the Rotterdam Study and validated their findings in 1539 participants from the HELIUS cohort, identifying several microbial taxonomic units associated with depressive symptoms [64]. The composition of the gut microbiome may therefore play a key role in depression.

### 4.2. Zinc Status Shapes the Gut Microbiota

Human evidence on zinc status and the gut microbiota remains limited. Chen et al. observed differences in inflammatory markers and microbial composition between zinc-deficient and control children, but the microbiome analysis involved only 26 zinc-deficient participants and 41 controls and cannot establish causality [65]. Most mechanistic evidence comes from animal models in which zinc availability is altered through dietary manipulation, supplementation, or deletion of zinc-transporter genes [66,67,68,69,70,71]. Collectively, these studies suggest that zinc availability can modify microbial composition under experimental conditions; they do not establish that comparable changes occur in humans or that such changes mediate depression.

At the species composition level, zinc deficiency alters the abundance of gut microbiota across all taxonomic levels. The impact of zinc deficiency on the gut microbiota extends beyond structural remodeling, directly impairing the core metabolic functions and physiological potential of the microbial community [71]. These findings underscore the essential role of zinc in maintaining normal gut microbiota function. As the primary metabolic end products of the gut microbiota, short-chain fatty acids exhibit synthesis levels that are significantly regulated by zinc availability. As summarized in Table 1, experimental manipulation of zinc availability altered several microbial taxa, including *Lactobacillus*, *Akkermansia*, *Coprococcus*, *Faecalibacterium*, and *Dialister*, together with changes in intestinal barrier proteins, inflammatory markers, and SCFA production [66,67,68,69,70,71,72,73]. Of particular relevance to depression, butyrate-producing taxa such as *Coprococcus* and *Faecalibacterium* have been associated with a higher quality of life, whereas reduced abundances of *Coprococcus* and *Dialister* have been reported in individuals with depression [63,64]. A study in poultry confirmed that zinc deficiency significantly reduces the production of acetate and hexanoate [72]. Studies in mice have revealed a time-dependent effect: short-term zinc deficiency increased acetate production, whereas prolonged deficiency led to a marked decrease in both valeric acid production and total short-chain fatty acid levels [73]. Functional genomic analyses further show that zinc deficiency broadly suppresses gene expression across a range of core metabolic pathways in the gut microbiota, including carbohydrate metabolism, glycan biosynthesis and metabolism, nucleotide metabolism, and amino acid metabolism [71,72,73]. These metabolic impairments are closely associated with host zinc absorption and inflammatory responses [66,67,69,70], suggesting that zinc-deficiency-induced disruption of the gut microbiota may increase the host’s risk of developing disease.

### 4.3. Zinc May Participate in Microbiota–Gut–Brain Communication

As outlined in Section 4.1 and Section 4.2, the gut microbiota plays a pivotal role in maintaining host physiological homeostasis, and its dysregulation is considered a significant mechanism in the pathogenesis of depression. At the same time, zinc not only participates directly in neurotransmitter synthesis and metabolism but also influences brain function and emotional states. It is therefore plausible that zinc affects the host’s emotional state through the gut microbiota. In a separate post-weaning mouse model, a zinc-deficient diet altered the intestinal microbiome and host metabolic phenotype [74]. The brains of these zinc-deficient mice showed signs of neuroinflammation, most directly evidenced by increased GFAP and IL-6 levels. Supplementation with a zinc amino acid complex partially restored microbial composition, intestinal pathology, and inflammatory cytokine levels. These findings suggest that zinc deficiency may disrupt gut–brain signaling by altering intestinal physiology, modifying microbiota composition, and elevating inflammatory markers. Although GFAP and IL-6 are not specific markers of depression, their elevation in this context warrants further investigation. In addition, zinc nanoparticle-fed mice exhibited enhanced 5-HT biosynthesis, transport, and receptor activation in the gut, alongside increased cerebral 5-HT levels via gut–brain communication [75]. Transplantation of human-derived microbiota provided direct evidence for the transferability of microbiota-associated effects. When microbiota from patients with major depressive disorder were transplanted into germ-free mice, the recipients developed depression-related behaviors and alterations in carbohydrate and amino acid metabolism [76].

Zinc deficiency-induced dysbiosis alters the production of microbial metabolites, including SCFAs and neuroactive substances. These metabolites influence brain function and emotional regulation via the vagus nerve, immune modulation, and neuroendocrine pathways. Reduced abundance of probiotic bacteria such as *Akkermansia muciniphila* is associated with increased depression risk, and supplementation with this species alleviates depressive symptoms induced by prenatal metal exposure, suggesting that certain gut microbiota components may protect against depression associated with zinc deficiency [77]. The interaction between maternal zinc status and gut microbiota also influences childhood depression scores, highlighting the pivotal role of the gut–brain axis in linking developmental zinc deficiency to depression [78]. Neither of these two cohort studies explored the underlying relationships in sufficient depth. It remains unclear, for example, whether alterations in the gut microbiota preceded the onset of depressive symptoms, and the potential contributions of children’s dietary patterns, family environment, antibiotic use, and other environmental exposures cannot be ruled out. Nevertheless, zinc is an essential micronutrient that plays important roles in neurobiological and systemic homeostasis; maintaining adequate zinc status may therefore be particularly relevant for individuals with depression or those at risk of zinc deficiency. Greater attention should therefore be directed toward evaluating diverse forms of zinc supplementation as potential strategies for preventing or correcting zinc deficiency.

Taken together, the available evidence supports a working model in which zinc-related neurobiological and intestinal pathways interact rather than operate independently. In the central nervous system, reduced zinc availability may attenuate GPR39–CREB/BDNF signaling, alter excitatory and inhibitory neurotransmission, and increase susceptibility to oxidative and neuroinflammatory stress. In parallel, altered intestinal zinc availability may impair epithelial barrier integrity and modify microbial community structure and metabolite production, including SCFAs, thereby influencing peripheral immune and neuroendocrine signaling. These gut-derived signals could further converge on central inflammatory and plasticity-related pathways, potentially contributing to depression-related phenotypes. This framework should be regarded as a working conceptual model rather than an established causal sequence, because the interactions among GPR39, BDNF, neuroinflammation, the gut microbiota, and SCFAs have not been examined within a single experimental framework or causally confirmed in humans. Future longitudinal and interventional studies should therefore jointly assess zinc status, microbial metabolites, inflammatory markers, and neuroplasticity-related outcomes to identify the pathways that mediate clinical responses.

## 5. Zinc Complex Supplementation Strategy

**Figure 3:**
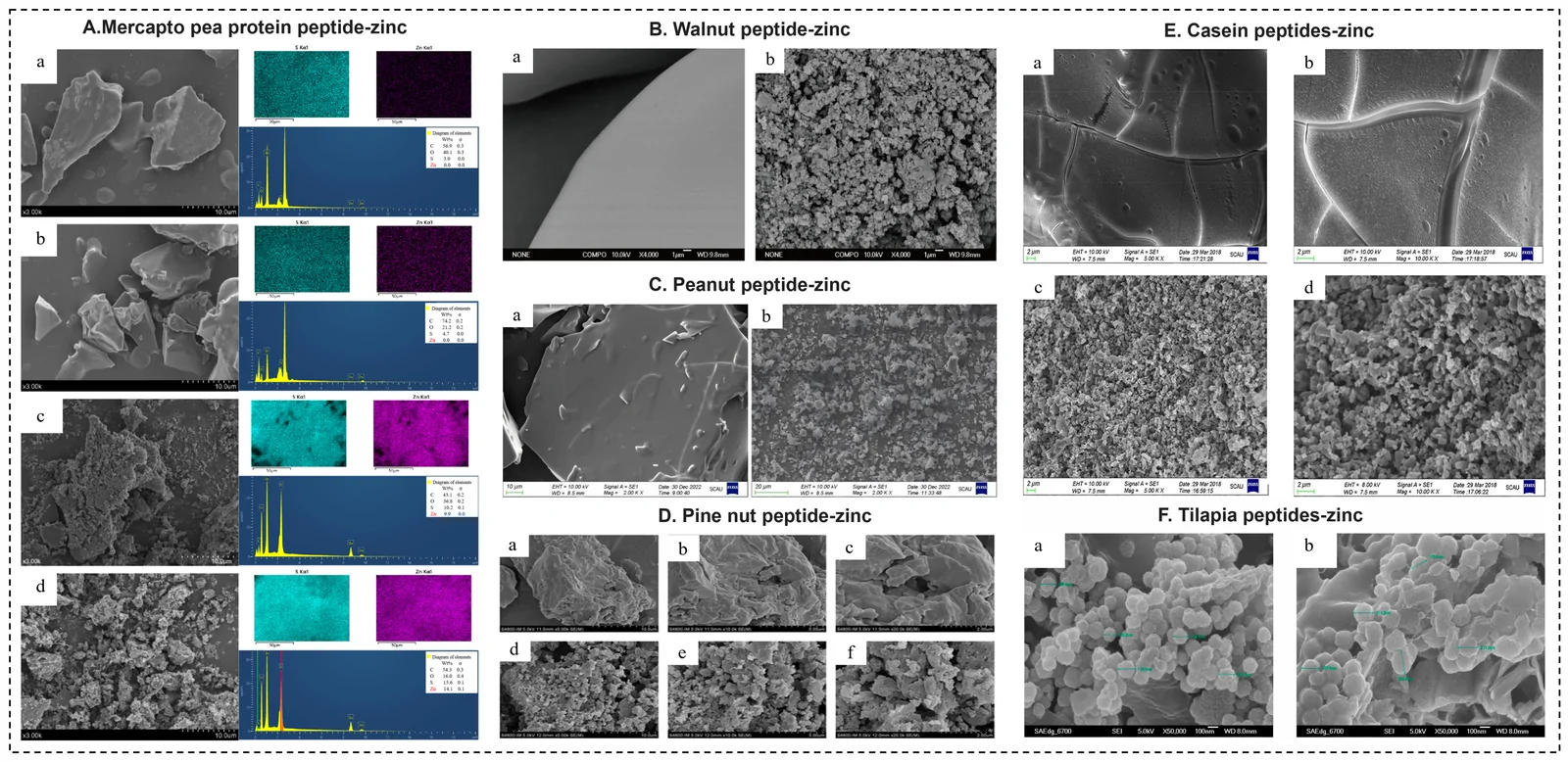
Typical structures of peptide-zinc, the resolution is 3000× (**Aa**–**Ad**), 4000× (**Ba**,**Bb**), 2000× (**Ca**,**Cb**), 1000× (**Da**–**Df**), 5000× (**Ea**–**Ed**), 50,000× (**Fa**,**Fb**). Ref: [80,83,85,86,87].

**Figure 4:**
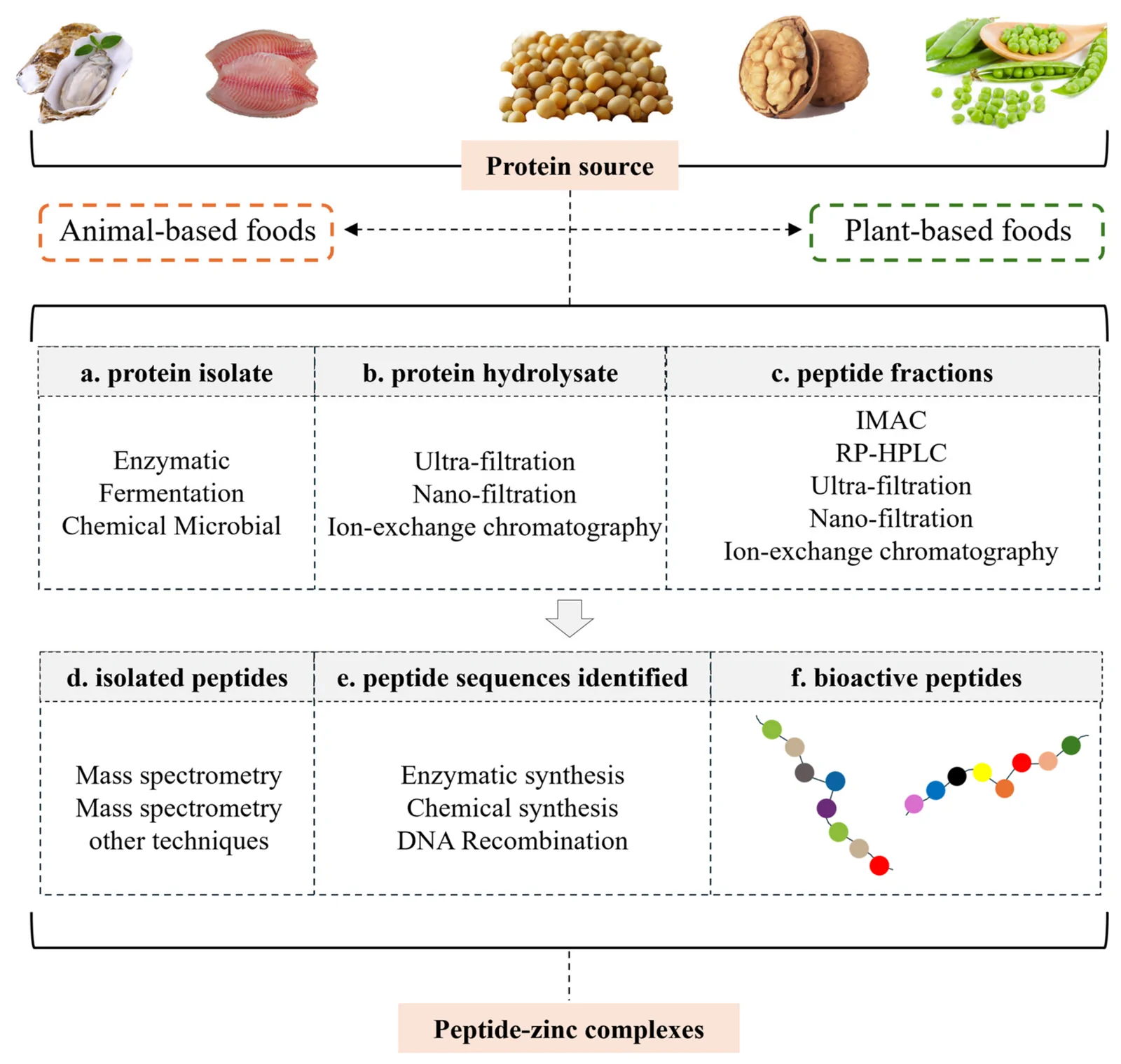
Generalized preparation workflow for peptide–zinc complexes. Specific hydrolysis, complexation, and purification conditions vary according to protein source and peptide characteristics.

**Figure 5:**
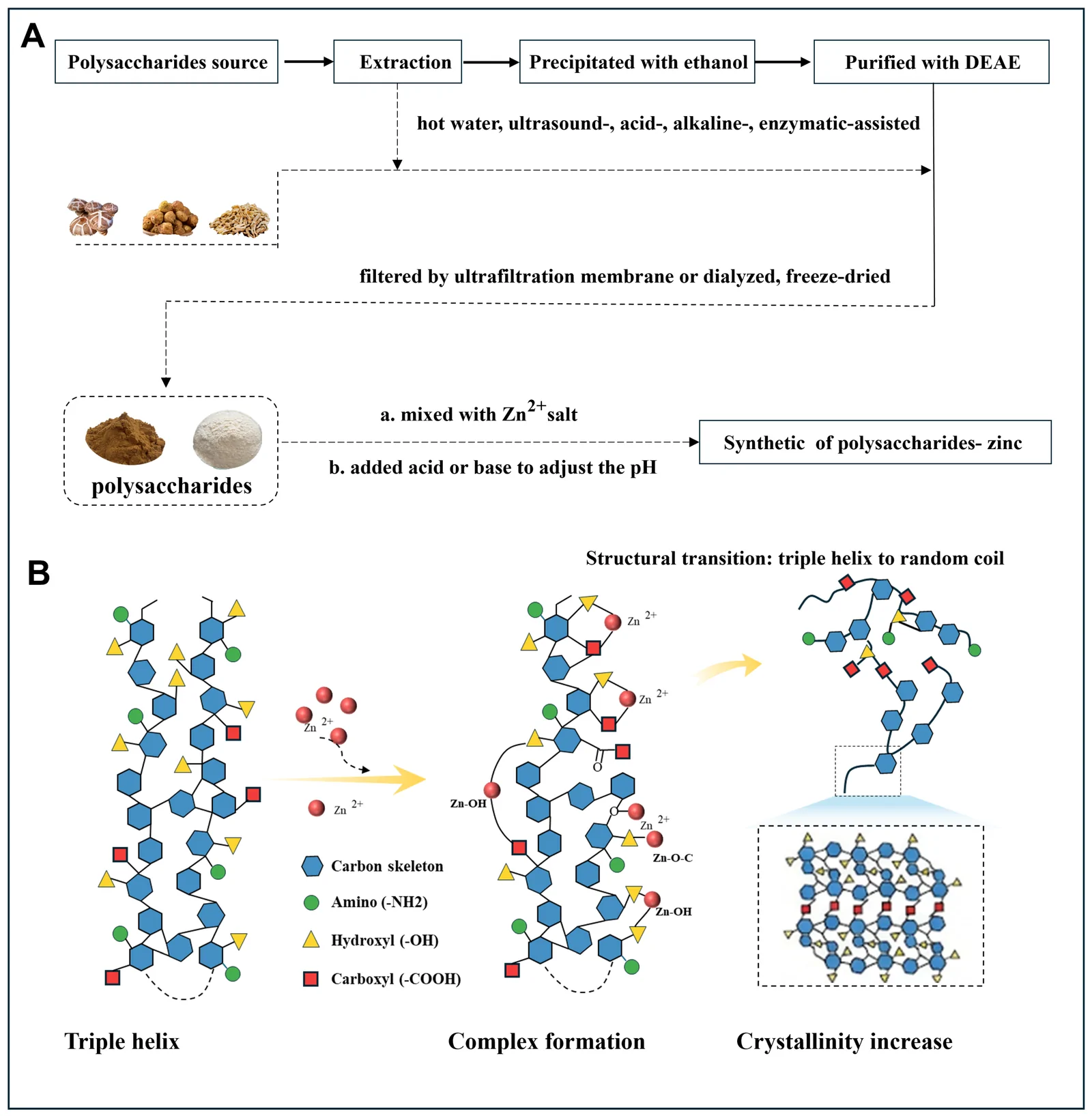
Generalized preparation workflow (**A**) and representative structural features of polysaccharide–zinc complexes (**B**).

**Figure 6:**
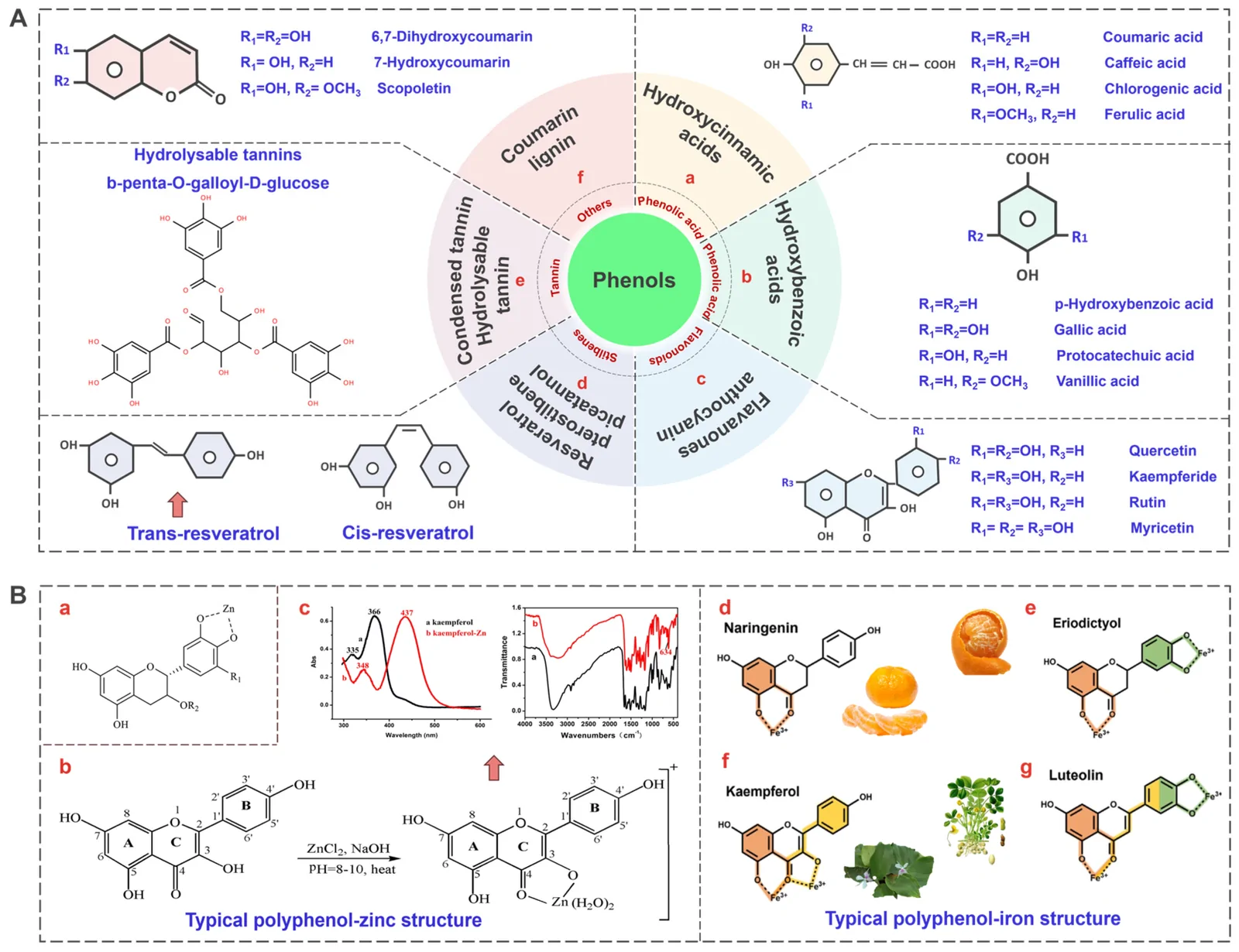
Common phenolic compounds (**A**). Typical polyphenol-zinc complexes (**Ba**–**Bg**). Ref: [96,97,98].

Given that zinc is an essential element, the following questions must be addressed: how might it be supplemented, and what food matrices should be selected? It is widely recognized that the primary obstacles to improving the bioavailability of zinc supplements typically arise at the gastrointestinal stage. Absorption rates can only be achieved when zinc is efficiently released from food or formulation matrices and maintained in a soluble or absorbable form. The exclusive use of inorganic zinc salts in supplements often results in low utilization, making it difficult to meet physiological requirements at practical intake levels. Concurrently, excessive zinc salt intake may pose potential health risks. Furthermore, inorganic zinc formulations are unstable under certain conditions, and prolonged use may irritate the intestinal environment and interfere with the absorption of other nutrients. Beyond the formulation itself, dietary antinutrients such as phytate and dietary fiber readily form insoluble complexes with zinc, thereby reducing its absorbable fraction. Divalent/multivalent metal ions such as iron and calcium, as well as certain proteins, can also inhibit zinc absorption through competitive or chelation effects [79]. Currently, multiple studies have shifted their focus from inorganic salts to organic complexation/chelation systems in order to enhance bioavailability. Common complex systems, as shown in Table 2 and Table 3 and Figure 3, Figure 4, Figure 5 and Figure 6, include peptide-zinc, polysaccharide-zinc, and polyphenol-zinc complexes [80,81,82,83,84,85,86,87,88,89,90,91,92,93,94,95,96,97,98].

### 5.1. Peptide-Zinc Complexes

Peptide-zinc complexes are food-derived zinc delivery systems formed by coordinating zinc ions with peptides obtained from enzymatic protein hydrolysis. As summarized in Table 2 and Figure 3 and Figure 4, their zinc-binding capacity is governed primarily by peptide size, charge, hydrophobicity, and the presence of coordinating residues, particularly histidine, cysteine, aspartic acid, and glutamic acid. Low-molecular-weight peptides generally provide accessible binding sites, while carboxyl, amino, imidazole, and thiol groups contribute to zinc coordination. These structural features improve zinc solubility and stability during gastrointestinal digestion relative to conventional inorganic zinc salts [98,99,100,101,102,103,104,105].

Experimental studies further indicate that peptide characteristics influence intestinal zinc uptake. In Caco-2 cell models, peptide charge and hydrophobicity affected zinc transport, with concomitant changes in PepT1 and ZnT1 expression [84]. Oyster-derived peptide–zinc complexes engage ZIP4-dependent and paracellular transport pathways and improve zinc-related metabolic outcomes in animal models [82]. Selected peptide–zinc complexes have also demonstrated antioxidant and cytoprotective effects in cellular systems, including increased antioxidant enzyme activity and reduced lipid peroxidation and apoptosis [104]. These findings indicate that peptide complexation may improve the gastrointestinal stability and intestinal availability of zinc.

### 5.2. Polysaccharide-Zinc Complexes

Polysaccharides contain abundant hydroxyl, carboxyl, and other oxygen-containing functional groups that can coordinate with zinc ions to form polysaccharide–zinc complexes. These interactions may improve zinc stability and confer additional biological properties, although evidence for enhanced zinc bioavailability remains predominantly preclinical. Polysaccharide–zinc complexes are generally prepared through aqueous complexation of polysaccharides with zinc salts, followed by separation and purification. Representative preparation and structural features are illustrated in Figure 5, while general preparation approaches are summarized in Table 3.

The source and molecular structure of the polysaccharide strongly influence zinc-binding capacity, complex stability, and biological activity. Studies involving chitosan, plant-derived polysaccharides, and soluble soybean polysaccharides indicate that hydroxyl, amino, and carboxyl groups participate in zinc coordination. Zinc binding may also alter polysaccharide conformation, crystallinity, particle morphology, and release behavior [106,107,108,109,110].

Polysaccharide–zinc complexes have demonstrated antioxidant, anti-inflammatory, immunomodulatory, antidiabetic, and other biological activities in cellular and animal models [88,89,90,91,92,93,94,95,110]. However, these outcomes vary according to polysaccharide source, molecular weight, zinc content, and experimental model. Human absorption, safety, and efficacy data remain insufficient, and no clinical evidence currently supports their use in depression.

Moreover, zinc ions adopt diverse coordination geometries within polysaccharide structures, forming bonds such as Zn-OH and Zn-O-C that contribute to the diversity and functional versatility of the complexes. Upon binding to zinc ions, polysaccharides form stable coordination structures and gain biomedical utility through changes in physical morphology, including the formation of nanoparticles or colloidal structures [108]. For example, the complex formed between *Anemarrhena asphodeloides* polysaccharides and zinc via Zn-O and Zn-OH coordination bonds retains a structure similar to that of the unmodified polysaccharide and significantly enhances immunomodulatory activity by activating NF-κB and MAPK signaling pathways [110].

In summary, polysaccharides are a promising class of zinc supplementation agents. They form coordination bonds with zinc ions through functional groups such as hydroxyl and carboxyl groups, yielding stable zinc-polysaccharide complexes. However, polysaccharides from different sources, including chitosan, pectin, and soybean polysaccharides, differ in zinc-binding capacity, complex stability, and biological function. These differences are closely tied to their molecular structures, functional group distributions, and molecular weights.

### 5.3. Polyphenol-Zinc Complex

Polyphenols are a class of natural organic compounds widely distributed in plants, primarily in fruits, roots, stems, and leaves. They are obtained from dietary sources such as fruits, vegetables, tea leaves, and Chinese medicinal herbs; over 8000 varieties have been identified to date. These compounds contain a benzene ring and one or more phenolic hydroxyl groups in their molecular structure and belong to the category of plant secondary metabolites. Polyphenols are classified into phenolic acids, flavonoids, resveratrol-type polyphenols, tannins, and other compounds (Figure 6A). Based on their carbon skeleton, they are further divided into hydroxybenzoic acid derivatives and hydroxycinnamic acid derivatives. Hydroxybenzoic acid derivatives feature a C6-C1 structure and include gallic acid, protocatechuic acid, and gentisic acid as classic examples. Hydroxycinnamic acid derivatives contain a C6-C3 structure and include chlorogenic acid, caffeic acid, and coumaric acid [111]. These two classes differ primarily in the R1, R2, and R3 substituents on the benzene ring [112]. Flavonoid polyphenols, resveratrol-type polyphenols, and highly polymerized polyphenols also serve as effective metal ligands owing to their abundance of active functional groups, particularly phenolic hydroxyl and carboxyl groups. In 2014, Wei and Guo identified these zinc-binding sites by nuclear magnetic resonance spectroscopy [112]. Similar coordination occurs in other polyphenol–metal complexes, including the polyphenol–iron complexes described by Zhang et al. [96]. Figure 6B shows representative structures of polyphenol–zinc and polyphenol–iron complexes.

Although polyphenols are potential zinc-binding agents, some studies have reported that they may impede zinc absorption. For example, grape seed extract reduces zinc absorption by obstructing transport across the apical membrane of intestinal cells [113]. The inhibitory effect of polyphenols on zinc absorption remains unsettled. Brnić et al. found that sorghum polyphenols suppressed zinc absorption in the presence of phytate but not in its absence [114]. Other polyphenols, however, promote zinc absorption. Quercetin, a representative polyphenol, forms novel complexes with zinc through diverse coordination modes, markedly enhancing the cellular uptake of both zinc and quercetin. These complexes localize around the cell nucleus and facilitate zinc transmembrane transport. They are also more effective at promoting biological activities such as apoptosis, suggesting that quercetin–zinc complexes may enhance the physiological functions of zinc by modulating intracellular signaling pathways [115]. Preclinical evidence further suggests that polyphenols may influence intestinal mucosal integrity and metal-transporter expression, but these mechanisms have not been established in humans [115]. In vitro and animal studies support potential antioxidant, anti-inflammatory, and zinc-delivery effects of selected polyphenol–zinc complexes [41,116]. Human comparative absorption studies are scarce, and the clinical relevance of these formulations to depression is unknown. The effects of polyphenol structure, complexation method, dose, and dietary context therefore require further investigation.

## 6. Probiotics and Zinc Bioavailability: Potential Mechanisms and Current Limitations

**Figure 7:**
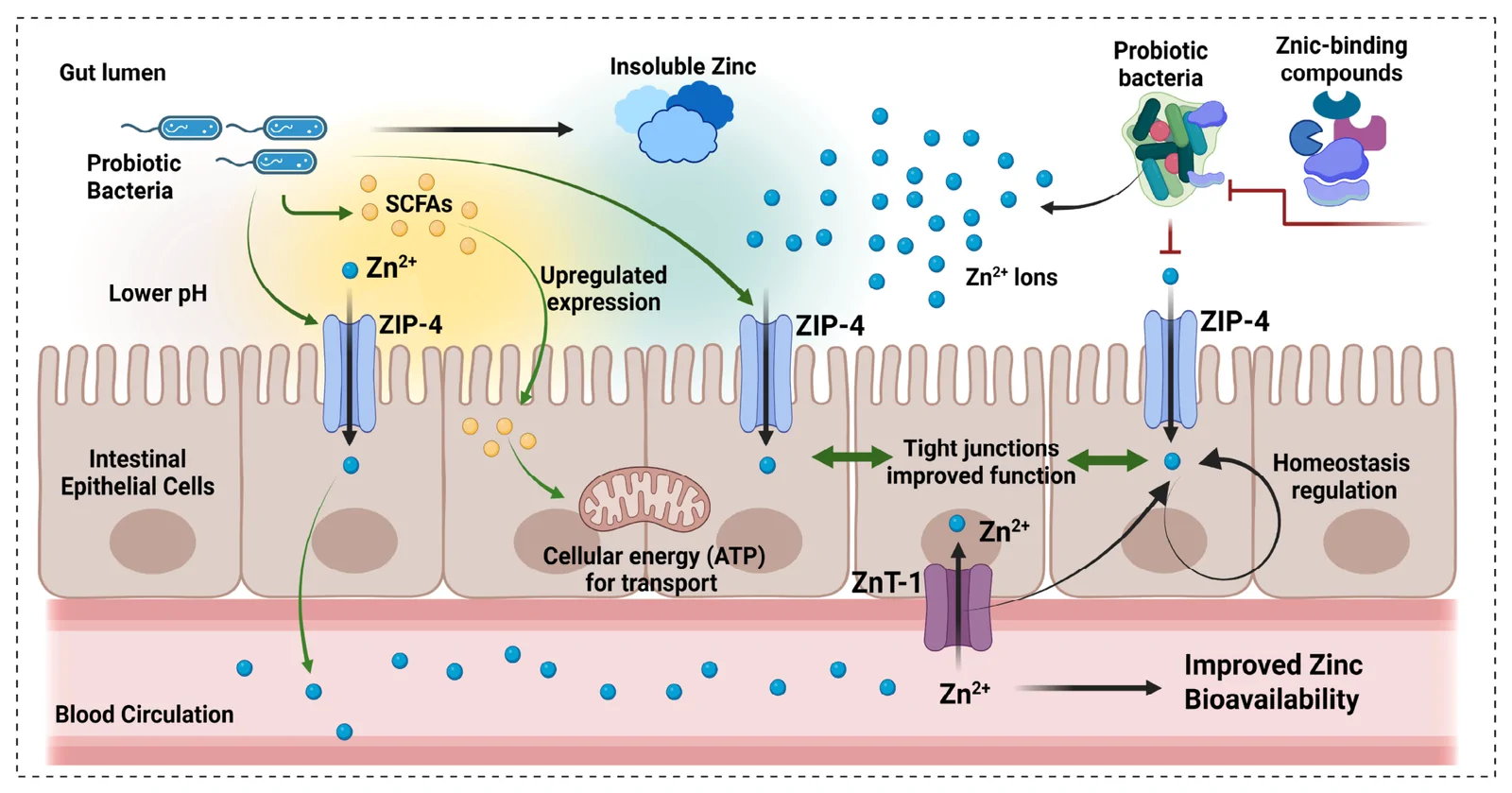
The schematic diagram illustrates the mechanism by which probiotics enhance zinc absorption.

It is well known that mineral levels in the body are primarily determined by dietary intake [117]. Mineral absorption occurs in the gut. Therefore, the gut microbiota plays a crucial role in this process. Probiotics primarily facilitate zinc absorption by improving the intestinal microenvironment of the host and modulating the expression of transport proteins (Figure 7). Currently, multiple studies indicate that probiotics can participate in host metabolism alongside minerals, thereby promoting improvements in health (Table 4).

### 6.1. Probiotics Modulate the Gut Microenvironment to Enhance Zinc Absorption

Probiotics enhance zinc absorption by regulating the intestinal microenvironment. Through fermentation, probiotics produce organic acids, particularly SCFAs such as lactic acid and acetic acid, which substantially lower intestinal pH. This acidification increases zinc solubility, keeping more zinc in its ionic form within the intestinal lumen and improving bioavailability—an effect especially important for a divalent cation whose solubility is strongly pH-dependent. SCFAs also act directly on intestinal epithelial cells: as an energy source for these cells, they enhance metabolic and transport functions, improving zinc absorption efficiency. In animal studies, co-administration of probiotics and zinc significantly increased intestinal villus height and mucosal thickness, strengthening the structural integrity of the intestine and facilitating the absorption of zinc and other minerals [125]. Probiotics further support zinc absorption by modulating the intestinal immune environment. They promote the growth of beneficial bacteria, suppress harmful bacteria, improve intestinal barrier function, and reduce inflammatory responses, collectively maintaining the gut’s capacity for nutrient uptake [126]. Probiotics also exhibit tolerance and biotransformation capabilities toward zinc. Certain lactic acid bacterial strains bind zinc ions through biosorption and bioaccumulation, converting them into forms such as nano-zinc oxide that reduce zinc toxicity while enhancing bioavailability [127,128]. These strains have been described as microbial nano-factories, offering new directions for the development of zinc formulations and the efficient utilization of zinc supplements.

Taken together, acidification of the intestinal lumen, production of microbial metabolites, maintenance of epithelial integrity, and modulation of microbial composition represent plausible mechanisms through which probiotics may influence zinc availability. Nevertheless, most supporting studies have used animal models or indirect markers such as intestinal morphology, inflammatory status, and transporter expression rather than fractional zinc absorption. These mechanisms therefore remain provisional, and their relative contribution is likely to vary among probiotic strains and host conditions.

### 6.2. Probiotics Influence Zinc Transporter Expression and Zinc Homeostasis

Probiotics have a significant regulatory effect on zinc transporter expression, promoting transmembrane zinc transport and maintaining zinc homeostasis. The zinc transporter family comprises two main categories: ZIP and ZnT, which regulate zinc uptake and efflux, respectively. Probiotics can modulate the expression of these zinc transporters through multiple mechanisms, thereby enhancing zinc bioavailability. For example, in weaned piglets, the combination of low-dose zinc oxide (AZO) supplementation and probiotics was found to significantly upregulate the expression of intestinal ZIP4 and DMT1, thereby enhancing zinc absorption and transport whilst reducing hepatic zinc accumulation and the associated oxidative damage [129]. This combination improved zinc bioavailability and reduced fecal zinc excretion, demonstrating the dual value of probiotics in regulating zinc transporter expression and environmental protection. Furthermore, introducing probiotics enhanced intestinal barrier function, as evidenced by increased expression of tight junction proteins, mucins, and antimicrobial peptides, as well as elevated gut microbial diversity and increased abundance of probiotic species such as *Lactobacillus*.

Probiotics contribute to zinc homeostasis by regulating zinc transporters, and they also participate in intestinal immunity and anti-inflammatory responses, demonstrating their immunomodulatory potential. A systematic review indicates that probiotics can enhance the absorption of minerals such as zinc, thereby alleviating deficiencies. They do this by regulating zinc transporter expression, optimizing the intestinal microenvironment and restoring intestinal barrier integrity [125]. Furthermore, probiotics increase the abundance of zinc transport-related genes such as zupT through their interaction with beneficial soil elements, thereby promoting active zinc transport even further. This highlights the multi-level role of probiotics in maintaining zinc homeostasis [130].

In animal models, combined supplementation with probiotics and zinc improves intestinal tissue structure by increasing villus height and mucosal thickness, which in turn enhances digestive absorption capacity and promotes the regulation of hormones such as ghrelin and incretin. Together, these effects contribute to the synergistic maintenance of zinc homeostasis and overall nutritional status [126]. Probiotics also influence the expression of the intestinal zinc transporter ZnT-1, regulating zinc efflux and distribution to prevent toxicity from zinc excess [130]. Their regulation of zinc homeostasis further synergizes with their antioxidant and anti-inflammatory effects, helping to mitigate the oxidative stress and inflammatory damage caused by zinc metabolic imbalance [131,132]. Taken together, probiotics maintain zinc homeostasis by regulating the expression of zinc transporters (including the ZIP and ZnT families), promoting transmembrane zinc transport, optimizing the intestinal microenvironment, enhancing intestinal barrier function, and coordinating systemic zinc absorption and excretion. Several limitations should be considered when interpreting this literature. First, the probiotic preparations differ substantially in species, strain identity, strain combinations, viability, dose, and intervention duration. Because probiotic effects are often strain-specific, findings obtained with one preparation cannot be generalized to other strains, products, or host populations. Second, zinc has been administered in inorganic, nanoparticulate, or microbially enriched forms, making it difficult to distinguish effects attributable to the probiotic, the zinc formulation, or their interaction. Third, most studies evaluated growth, intestinal morphology, antioxidant status, inflammatory markers, or general health outcomes rather than fractional zinc absorption, systemic zinc retention, or depression-relevant neurobehavioral outcomes. Notably, none of the studies summarized in Table 4 used a validated depression model or examined whether a probiotic–zinc combination modifies depressive-like behavior, GPR39-CREB/BDNF signaling, neuroinflammation, hypothalamic–pituitary–adrenal axis activity, or microbiota-derived neuroactive metabolites. The only human study summarized in Table 4 involved infants with acute gastroenteritis and was not designed to assess zinc absorption or mental-health outcomes. Major knowledge gaps therefore include the absence of strain-resolved comparative studies, factorial designs that separate the independent and interactive effects of probiotics and zinc, dose–response analyses, stratification according to baseline zinc status, and long-term safety assessments.

## 7. Conclusions and Outlook

Over the past decade, understanding of the biological roles of zinc in the nervous system has continued to advance. As an essential micronutrient, zinc contributes to the regulation of neurotransmission, synaptic plasticity, neurogenesis, neuroimmune homeostasis, and stress responses. Animal studies further indicate that disruption of zinc homeostasis is frequently accompanied by depression-related behavioral and neurobiological alterations. Nevertheless, an important translational challenge remains: improving zinc bioavailability in a clinically effective, sustainable, and individualized manner does not necessarily translate into consistent improvements in neural function or depressive symptoms. Zinc homeostasis cannot be reduced to a simple model of insufficient intake corrected by dietary replacement; it encompasses gastrointestinal absorption, systemic transport, intracellular trafficking, ligand-binding speciation, and dynamic redistribution among the gut, immune system, and central nervous system. Although advances in zinc metabolism research have laid the groundwork for optimizing supplementation strategies, many of these processes remain incompletely understood. The evidence base is therefore uneven: mechanistic support is strongest in cellular and animal studies, whereas human data are limited mainly to observational associations and small trials of conventional zinc salts.

Any consideration of zinc as an adjunctive intervention must account for the elemental zinc dose, chemical form, duration of use, total zinc exposure, and individual safety profile. Small randomized controlled trials in patients with major depressive disorder have typically administered 25 mg of elemental zinc per day for approximately 12 weeks alongside antidepressant treatment [133,134]. This dose, however, reflects what has been evaluated in clinical trials rather than an established therapeutic recommendation. The available studies have generally been small and heterogeneous, lacking adequate dose–response comparisons, long-term follow-up, and consistent stratification by baseline zinc status [135]. Because zinc salts and zinc complexes contain different proportions of elemental zinc, research reports and product labels should clearly distinguish the amount of elemental zinc from the total mass of the zinc-containing compound. Peptide–zinc, polysaccharide–zinc, and polyphenol–zinc complexes may improve physicochemical stability or intestinal availability. Whether they confer greater clinical benefit than conventional zinc salts in the management of depression, however, requires further confirmation.

Safety considerations are equally important, because habitual total zinc intake includes zinc from food, dietary supplements, and other zinc-containing products. The tolerable upper intake level for zinc in adults is 40 mg/day in the United States and 25 mg/day in Europe [136,137]. Excessive or prolonged zinc intake may cause gastrointestinal symptoms and interfere with copper absorption, potentially resulting in copper deficiency, hematological abnormalities, altered immune function, and reduced high-density lipoprotein cholesterol concentrations. Zinc may also interact with quinolone and tetracycline antibiotics, penicillamine, thiazide diuretics, and high-dose iron supplements [136]. Although several small trials have combined zinc with selective serotonin reuptake inhibitors or imipramine, these studies were not designed to evaluate pharmacokinetic interactions, rare adverse events, or long-term safety differences across antidepressant classes.

Future research should therefore address both mechanistic and clinical uncertainties. At the mechanistic level, studies are needed to determine how zinc deficiency alters microbial metabolic networks, including short-chain fatty acids, tryptophan–kynurenine metabolites, bile acid profiles, and microbially derived neuroactive compounds. Studies should also clarify how these changes influence vagal signaling, hypothalamic–pituitary–adrenal axis activity, peripheral inflammation, and blood–brain barrier integrity. At the clinical level, adequately powered randomized controlled trials should report the elemental zinc dose, chemical formulation, total dietary zinc exposure, adherence, adverse events, and concomitant medications. They should also prospectively evaluate whether baseline zinc status, inflammatory characteristics, age, nutritional risk, comorbidities, and depressive phenotype act as effect modifiers. Such studies may clarify the multidimensional contribution of micronutrients to brain health and establish a more rigorous scientific basis for the prevention, assessment, and adjunctive management of depression.

## Figures and Tables

**Table 1:** A range of animal models to demonstrate the effects of zinc on the host gut microbiota.

Subjects	Experimental Model	Change of Zinc	Key Findings	Ref
C57BL/6 J mice	Control: zinc adequate Model: lipopolysaccharide Treat: low dose: 5.13 mg/kg medium dose: 10.26 mg/kg high dose: 30.78 mg/kg	-	Treat group vs. control and model group ZO-1, Occludin, Claudin-1 and JAM-A ↑, MLCK ↓ *Bacteroidetes*, *Firmicutes*, *Verrucomicrobia*, *Parabacteroides*, *Lactobacillus*, and *Akkermansia* ↑, *Proteobacteria* and *Enterobacter* ↓	[66]
BALB/c mice	Zinc supplementation was achieved through drinking water, with all groups receiving standardized food (containing 25 mg/kg of zinc). control: <0.1 mg/mL treat: 250, 50, or 12 mg/kg	Zn-supplemented vs. control: whole blood, spleen, and feces Zn ↑	Treat group vs. control group *Clostridiaceae* ↑, *Lactobacillaceae* ↓, *Enterobacteriaceae* ↓ *Candidatus arthromitus* ↓, *Lactobacillus* ↓ IL-6, IL-1β and Nos2 ↓ ZnT2 ↑, ZIP4 ↓	[67]
Sprague Dawley rats	Adequate: 35–38 μg/g deficient: 1 μg/g	Adequate vs. deficient: serum, liver, testis, feces Zn ↑ No difference in zinc content between the ileum, caecum, and colon	Adequate group vs. deficient group *Lachnospiraceae nK4a136*, *Eubacterium coprostanoligenes* ↑; *Ruminococcus gauvreauii* ↓	[68]
Arbor Acres broiler chickens	Conventional ZnO group, nano-ZnO group: 82 nm and 21 nm	No significant difference in residual zinc content in broiler chicken excreta	Nano-ZnO group vs. conventional ZnO group: thymus index, spleen index, metallothionein, superoxide dismutase, and lysozyme ↑ IL-1β and TNF-α ↓, IL-2, IFN-γ ↑ unidentified_*Lachnospiraceae*, *Blautia*, *Lachnoclostridium*, unidentified_*Erysipelotrichaceae*, and *Intestinimonas*↑	[69]
C57BL/6 J mice	All genetically modified mice obtained will be fed a semi-purified diet containing 30 mg zinc per kilogram, including ZnT7+/+, ZnT7+/− and ZnT7−/− strains	-	ZnT7−/− group vs. ZnT +/+ group: ZIP4 ↑, ZIP2 ↓ *Allobaculum* ↑	[70]
Landrace piglets	Supplemented with 40 or 110 ppm zinc as zinc oxide, 110 ppm as Zn-Lysinate, or 2500 ppm as zinc oxide	2500 ppm vs. 40 ppm Colon, jejunal tissue, liver, kidney, pancreas, bone Zn ↑	2500 ppm group vs. 40 ppm group: ZnT1, MT1a, MT2B ↑ and ZiP4 ↓ *Bacteroides*, *Parabacteroides*, *Collinsella*, *Acetivibrio*, *Blautia*, *Coprococcus*, *Faecalibacterium*, *Subdoligranulum*, *Holdemania*, *Intestinimonas*, *Lachnoclostridium*, *Pseudoflavonifractor* ↑, *Megasphaera*, *Dialister*, *Acidaminococcus*, *Ruminococcus*, *Treponema* ↓ metal ion binding, penicillin-binding, β-lactam antibiotic catabolism ↑, carbohydrate metabolic processes, pectinesterase, α-glucuronidase activity, α-l-arabinofuranosidase ↓ acetate, propionate, butyrate ↓	[71]

- Not detected, ↑ Increase, ↓ Decrease.

**Table 2:** The specific functions and properties of peptide-zinc complexes.

Sources of Peptides	Hydrolases	Interaction Forces	Biomarker	Conclusion	Ref
Pea protein peptides (CMP)	Complex protease	Carboxyl, amino and thiol groups	IEC-6 cell	The zinc absorption rate of CMP-Zn via receptor-mediated endocytosis in IEC-6 cells was 32.59% ± 2.85%, which was 2.1 times that of ZnSO_4_	[80]
Walnut Peptide (WP)	Alkaline protease	Hydrophobic interactions, hydrogen bonds and electrostatic forces	Caco-2 cell	The WP-Zn demonstrates superior thermal, acid-base, and gastrointestinal digestive stability compared to ZnSO_4_	[81]
Oyster Peptide (OP)	Alkaline protease, trypsin, neutral protease	The chemical bond between oxygen and nitrogen	Caco-2 cell	OPZs can modulate the basal expression of key zinc transporters such as ZIP4, ZnT1 and MT1, as well as the peptide transporter PePT1	[82]
Tilapia skin peptides (TSP)	Alcalase, trypsin	Carboxyl group, amino group	Caco-2 cell	GFLGSP can be transported across a monolayer of Caco-2 cells, with a Papp value of (2.17 ± 0.23) × 10^−6^ cm/s. Furthermore, the paracellular pathway via tight junctions may participate in the transport process of the peptide substance GFLGSP	[83]
Soybean peptides	Trypsin, pepsin	Carboxyl, carbonyl and amino groups	Caco-2 cell	Negatively charged complexes enhance the activity of zinc-dependent enzymes, with PepT1 and ZnT1 exhibiting higher expression levels than other peptide-zinc complexes	[84]
Casein	Trypsin	Carboxyl, carbonyl and amide bonds	Caco-2 cell	Highly bioactive zinc-chelating peptides extracted from casein significantly enhance zinc transport in Caco-2 cell monolayers	[85]

**Table 3:** The specific functions and properties of polysaccharide-zinc complexes.

Zinc Reagent	Polysaccharide Type	Zinc Content	Biological Activity	Ref
ZnCl_2_	*Fritillariauss uriensis*	7.2%	Antioxidant	[88]
ZnSO_4_	*Garlic*	5.15%	Antioxidant	[89]
ZnSO_4_	*Ginger peel*	21.2 mg/g	Anti-inflammatory	[90]
ZnSO_4_	*Cucurbita moschata*	23.2 mg/g	Anti-inflammatory	[91]
ZnCl_2_	*Dioscorea opposita Thunb*	24.9%	Anti-diabetic	[92]
ZnAc	*Prunella vulgaris*	27.0 mg/g	Anti-tumor	[93]
ZnCl_2_	*Hohenbuehelia serotina*	7.1%	Antioxidant	[94]
ZnSO_4_	*Athelia rolfsii*	54.6%	Antioxidant	[95]

**Table 4:** Preclinical and clinical studies evaluating combined probiotic and zinc interventions.

Probiotic	Type of Study	Nr. of Subjects	Intervention	Outcome	Ref.
*Lactobacillus plantarum* *Lactobacillus acidophilus* *Lactobacillus delbrueckii* subsp. *bulgaricus*, *Bifidobacterium bifidum* *Lactobacillus rhamnosus* *Enterococcus faecium* *Streptococcus salivarius* subsp. *thermophilus* *Aspergillus oryza* *Candida pintolopesii*	In vivo on broiler chicks	32	42-day	The combined effect of zinc and probiotic mixtures demonstrated superiority in improving intestinal growth performance and tissue morphology	[118]
*Lactobacillus acidophilus* *Saccharomyces cerevisiae*	In vivo on Wistar rats	60	30-day	Selenium/zinc-enriched probiotic feed supplements represent a potential nutritional feed for subtropical and tropical regions, mitigating the detrimental effects of prolonged heat stress	[119]
*Lactobacillus acidophilus* *Saccharomyces cerevisiae*	In vivo on Wistar rats	36	40-day	Zinc-enriched probiotic supplementation enhances growth performance in Wistar rats under high-temperature conditions by improving antioxidant status, immune function, relevant gene expression, and intestinal morphological characteristics	[120]
*Lactobacillus plantarum*	In vivo on C57BL/6 mice	68	7-day	Zinc-enriched *Lactobacillus plantarum* also demonstrated superior protective effects against colitis in mice compared to ZnSO_4_	[121]
*Lactobacillus plantarum*	In vivo on ICR mice	32	28-day	Selenium and zinc-enriched strains enhanced antioxidant capacity in mouse blood and improved utilization of selenoaminoacid	[122]
*Lactobacillus reuteri*	In vivo on infants with acute gastroenteritis	51	5-day	Among well-nourished infants and young children with acute diarrhea who are not hospitalized, oral rehydration salts supplemented with probiotics and zinc demonstrate favorable efficacy in treating acute diarrhoea	[123]
*Bacillus licheniformis*	In vivo on chicken	420	35-day	Zinc and *Bacillus licheniformis* enhanced the body weight, carcass characteristics, and meat quality traits of broiler chickens	[124]

## Data Availability

No new data were created or analyzed in this study. Data sharing is not applicable to this article.
